# Surgery for Crohn’s disease: upfront or last resort?

**DOI:** 10.1093/gastro/goac063

**Published:** 2022-11-09

**Authors:** U Ahmed Ali, Ravi P Kiran

**Affiliations:** Division of Colorectal Surgery, New York–Presbyterian/Columbia University Medical Center, New York, USA; Division of Colorectal Surgery, New York–Presbyterian/Columbia University Medical Center, New York, USA

**Keywords:** surgery, timing, Crohn’s disease, outcome

## Abstract

Crohn’s disease (CD) can involve the entire gastrointestinal tract from the mouth to the anus and can lead to a constellation of symptoms. With the advancement of effective medical treatments for CD, a tendency has emerged to consider surgical treatment as a last resort. This potentially has the disadvantage of delaying surgery and if it fails might leave patients sicker, less well nourished, and with more severe complications. As with most non-malignant diseases, the choice of surgery vs medical treatment is a patient’s personal preference under the guidance of the treating physician, except in extreme situations where surgery might be the only option. In this article, we will discuss the available evidence regarding the optimal timing of surgery in CD, focusing on whether early surgery can bring benefits in terms of disease control, symptom relief, and quality of life.

## Introduction

It is widely acknowledged that Crohn’s disease (CD) is characterized by a progressive course, starting with mucosal inflammation, and if left untreated can advance to transmural involvement with risk of perforation, abscesses, fistulas, and fibrotic stenosis [1–3]. Unfortunately, no curative treatment for CD exists as of yet [4]. Therefore, any intervention aims to provide symptom relief and increase the quality of life (QOL).

Medical treatment aimed at reducing inflammation is often the first step in the treatment of patients with CD. Conventionally, corticosteroids are commonly used for induction, while thiopurines and methotrexate can be used for maintenance [5]. In recent decades, new biological agents have been developed that have significantly improved our ability to control symptoms [6]. Antitumor necrosis factor (TNF) biologics (such as infliximab, adalimumab, and certolizumab) alone or in combination with other agents (e.g. azathioprine) are effective for both induction and maintenance [5, 7]. Newer biologics, such as vedolizumab and ustekinumab, are also playing an increasingly important role in the management of CD [8].

Current evidence suggests that these new treatment modalities have resulted in a significant decrease in the rate of surgery requirements over the last decades [9–13], although the exact extent is still up for debate [14]. It is clear, however, that even in the era of biologics, a considerable proportion of patients still require surgery in the course of their disease [15–18]. There are several indications for surgery in CD, with ileocolic resection being the most common procedure [19]. Most patients are operated on due to failure of medical management. This is often due to the persistence of symptoms or progression of disease despite (optimal) medical management. It might also be due to the occurrence of unacceptable complications of medical treatment necessitating cessation or the inability to wean off steroids, also known as steroid dependency. Additionally, disease-specific complications, such as abscesses, fistulae, hemorrhage, and obstruction, might require (urgent) surgical treatment in a substantial proportion of patients. Finally, neoplastic changes due to chronic inflammation, i.e. dysplasia and cancer, also require surgery in most cases.

Optimal management of CD mandates optimal timing of both medical and surgical treatment modalities. Several studies have suggested that undue delay with the use of biologics, for example, might be responsible for suboptimal management and failure to achieve adequate disease control [20–22]. Similarly, delays in surgery might result in the need for extensive resections and might increase the risk of complications, due to the deterioration of inflammatory status, malnourishment of patients, and the presence of perforating disease manifestations such as fistulas or abscesses [23].

In this article, we will discuss the available evidence regarding the optimal timing for surgery in CD, focusing on whether early surgery can bring benefits in terms of disease control, symptom relief, and QOL.

## How to define the timing of surgery?

It is important to consider the various approaches that can be used to define the timing of surgery in CD. One approach is to use the time period from diagnosis or symptom onset to surgery. While this might seem like a straightforward approach, it has the important limitation that the time span of disease progression can differ significantly between patients. Also, this measure is less informative regarding the exact indication for surgery at any certain time. Additionally, the exact moment of symptom onset might be difficult to recall for patients, especially in cases with a protracted history prior to diagnosis. All make drawing conclusions and formulating recommendations for individual patients quite challenging.

Another approach is to study the outcome of surgery at the initial time of diagnosis compared with surgery performed at a later stage. This approach has been adopted by several studies discussed below. It should be noted that, in retrospective studies, this is a dichotomization of the time-span definition described above and suffers from similar limitations. It also suffers from a strong selection bias, since not all patients have indications for surgery at the time of diagnosis. It does have the advantage in the research setting of increasing the ease of interpretation since a direct comparison between groups can be performed rather than more complicated analysis techniques such as regression analysis or survival analysis.

A different approach that might be more beneficial in clinical practice is the use of the disease presentation to establish criteria for early vs late surgical intervention. This could be a combination of disease severity scores, the presence of complications, and response (or lack thereof) to medical management. To our knowledge, no such formal or validated system exists. Devising such criteria might provide a valuable tool to allow the prospective evaluation of whether early surgery does indeed offer any advantages to CD patients. In this paper, we will also attempt to provide recommendations using such an approach to aid in clinical practice.

## Surgery as a primary treatment for CD

Primary treatment for CD with surgery is uncommon in clinical practice. Few studies exist that report the outcomes of this approach. A 2007 retrospective study described a cohort of 207 patients with CD that underwent ileocolic resection [24]. Of these, 83 patients had surgery for acute CD; the diagnosis was made per-operatively and was confirmed by histopathology (early surgery). None of the patients received medical treatment before surgery. The other 142 patients underwent surgery on average 54 months after diagnosis (late surgery). All of them received specific CD treatment prior to surgery. Indications for surgery were either refractoriness to medical treatment or complications. There was no difference between the two study groups in regard to penetrating vs non-penetrating disease. The early surgery group was associated with a reduced risk of clinical recurrence and reduced need for immunosuppressants (*P *=* *0.01 and *P *=* *0.05, respectively). No difference was seen regarding the risk of surgical recurrence (*P *=* *0.53) after a median duration of 147 months (range 12–534). In multivariate analysis, early surgery was the only independent predictor for reduced risk of clinical recurrence (hazard ratio [HR]* *=* *0.57; 95% confidence interval [CI] 0.35–0.92; *P *=* *0.02), but was not associated with the other two study outcomes.

A study from 2009 addressed this question in a slightly different manner. In a group of 490 patients with CD recruited between 1980 and 2005, 115 had a diagnosis of CD at the surgery for acute abdomen (“early surgery” group) [25]. These were compared with the remainder of the group that included patients managed medically as well as those who underwent surgery in the course of the disease (“clinical diagnosis” group). Patients in the early surgery group had more stricturing and penetrating disease at presentation and the disease was more frequently present in the ileum and less in the colon. The mean follow-up was 168 months for the early surgery and 104 months for the clinical diagnosis group. During follow-up, patients in the early surgery group used fewer salicylates (odds ratio [OR]* *=* *0.03; 95% CI 0–0.6; *P *<* *0.002), steroids (OR = 0.3; 95% CI 0.2–0.5; *P *<* *0.0001), and conventional immunosuppressive drugs (OR = 0.6; 95% CI 0.3–0.9; *P *<* *0.04). The study also reported a reduced probability of intestinal resection in the early surgery group. However, it should be noted that they compared the rate of a second surgery (reoperation rate) in the early surgery group with the first surgery rate in the late group.

A Korean study including 243 patients compared 120 patients who underwent segmental bowel resection in the period of 1 month before or after diagnosis of CD (“early group”) and 123 patients who received surgery at a later date (“late surgery”) [26]. The median time to surgery from the onset of symptoms was 2 months vs 39 months, respectively. Notably, patients treated with immunomodulators or biologics before surgery were excluded from both groups. The median follow-up was over 8 years. The early group included significantly more patients operated on due to stricturing (34% vs 17%) and penetrating disease (42% vs 20%). Late surgery was associated with the increased post-operative use of biologics (OR = 1.95; 95% CI 1.11–3.34), but not with the risk of reoperation (OR = 1.47; 95% CI 0.74–2.90).

While the definition of early surgery in the abovementioned studies is clinically not practical (i.e. definitive diagnosis was mostly not known before surgery), it arguably offers a unique insight into whether outright surgery for CD has any potential benefits. The fact that patients in the early groups all underwent (semi-)urgent surgery seems to indicate a more severe disease presentation in this group. This can confound outcomes against early surgery. Nonetheless, all studies show that early surgery has no harm in terms of disease progression and might offer some advantages.

## Surgery in the course of CD

A number of studies have looked at studying the effect of timing of surgery at different stages of CD on disease outcome. The most important study on this topic is the LIR! C trial [27]. In this trial, 143 patients with limited active disease of the terminal ileum (<40 cm) were included. Patients were randomized to receive either surgery or infliximab treatment after failure of conventional treatment (at least 3 months of glucocorticosteroids, thiopurines, or methotrexate). The primary outcome was QOL and secondary outcomes included complications and body image. After a median follow-up of 4 years, 37% of patients initially randomized to infliximab received ileocecal resection, while 26% of the surgical group required infliximab treatment. No differences between treatment groups were seen in the QOL and complications. Long-term follow-up showed that the duration of treatment effect, defined as the time without the need for additional CD-related treatment was similar between the two groups [28]. The authors conclude that these results support ileocaecal resection as a reasonable treatment option in patients with limited CD that have failed conventional treatment.

A second randomized–controlled trial (RCT) randomized patients with ileocecal CD to either medical treatment or primary ileocecal resection [29]. The medical treatment consisted of induction with budesonide and thereafter maintenance treatment with azathioprine. Unfortunately, the study was prematurely terminated due to accrual difficulties and changes in clinical practice. Analysis of the 36 included patients showed improved QOL and general health in patients receiving early surgery after 1 year of follow-up, while no differences were seen for disease activity during follow-up.

Several retrospective studies also examined this topic. A recent study including 307 consecutive patients recruited between 1994 and 2018 performed a risk factor analysis for post-operative complications [30]. Of all the variables included, the time interval between diagnosis of CD (OR = 1.1 per year; 95% CI 1.03–1.17) and preoperative steroid use were the only independent risk factors for major surgical complications. Another 2018 retrospective study compared short-term post-operative outcomes of 46 patients who received elective surgery within 5 years of diagnosis with 77 who received it after 5 years [31]. The latter group had a higher rate of overall surgical complications (40% vs 30%, *P *=* *0.01) including a higher rate of reoperations (21% vs 7%, *P *=* *0.003) and anastomotic leakage (13% vs 4%, *P *=* *0.01). The overall rate of medical complications was also higher in the late surgery group (25% vs 15%, *P *=* *0.02). These data support the notion that undue delay of surgery can be associated with more complex operations and higher rates of post-operative complications.

A German study also evaluated patients who underwent ileocolic resection for CD and included a total of 103 patients [32]. Patients were divided into those who received primary resection without previous medical therapy (*n *=* *29) and those who were treated medically first (*n *=* *74). The median time from diagnosis to primary surgery was 15.6 months compared with 85.8 months for the groups, respectively. The primary end point was the need for anti-inflammatory drugs in the 2* *years following surgery. Patients in the medical treatment first group showed increased rates of disease in the upper gastrointestinal tract (6.9% vs 20.3%) and perianal involvement (6.9% vs 27.0%) at initial presentation. Patients requiring initial medical treatment had a higher rate of medical therapeutics 2 years after surgery (38% vs 78%, *P *<* *0.001). Post-operative complications were similar between the two groups. These findings might be due to a selection bias since patients with the more generalized disease might have been selected for medical rather than surgical treatment.

A study from Hungary included 506 patients with CD recruited from 1977 to 2008 [33]. The authors identified patients who received limited resection within 12 months of diagnosis as the early surgery group. Based on logistic regression and propensity score matching, they concluded that early surgery is associated with benefits in terms of reducing the risk of surgery and decreased overall exposure to steroids and biologics. However, the study had serious methodological issues, such as confusing outcomes and risk factors in logistic regression, making results unreliable.

An Australian cohort study of CD patients with ileocolonic disease compared 42 patients receiving early surgery with 115 who received initial medical therapy [34]. Median follow-up was 67 and 97 months for the two groups, respectively. Patients in the medical therapy group had less stricturing and less penetrating disease at presentation. Patients receiving early surgery had fewer hospital admissions (1 vs 3, *P *=* *0.01) and a lower need for biologics post-operatively (33% vs 60%, *P *=* *0.004).

Another study examined the issue of timing in cases of acute complications in CD [35]. This study described 112 patients receiving ileocolonic resection due to acute complications of CD, including abscesses, fistulae, and microperforations. Patients were divided into two groups: early surgery defined as resection within the first 7 days of presentation vs delayed surgery defined as >7 days after presentation. The median time to surgery was 3 days in the early group vs 23 days of the “cool-off” period in the delayed group. No differences were seen between the two groups in surgery characteristics (laparoscopy rate, conversion, and diversion), 30-day post-operative complication rate (25% vs 17%), and readmission (6% vs 5%). After a median follow-up of 14 months, there was no difference in the rate of subsequent CD-related intestinal resection (4% vs 5%). The authors concluded that including a long “cool-off” period in this group of patients did not necessarily result in improved immediate or long-term outcomes. Needless to say, this group of patients does represent a group of patients with a higher surgical risk due to the perforating nature of the disease when compared with outcomes of patients operated on electively [21].

It should be noted that most studies discussed above are retrospective and suffer from selection bias. The patients in the early surgery group all underwent surgery probably due to a significantly different presentation than patients who were managed medically (bias by indication). Patients undergoing early surgery may a more severe presentation with more complications. Direct comparison of these groups can be challenging since the underlying disease pathophysiology and associated progression might be different. Additionally, there is a lot of heterogeneity in the definition of early surgery and the control group used. Nonetheless, the body of evidence does suggest that early surgery is safe and feasible, and might have some advantages compared with surgery at a later stage.

## Combination of biologics and surgery: is there an optimal mix?

### Combining medical and surgical treatment: biologics prior to surgery

None of the above studies has examined the influence of biologics on the outcome of surgery. The LIR!C trial, for example, has considered medical failure to be the failure of conventional treatment without preoperative use of biologics. This begs the question of whether a combination of timely biologics and well-timed surgery might result in a synergic effect further improving the outcomes for patients. Several studies show that delay of treatment in CD (either medical or surgical) is associated with worsening of outcomes. The International Program to develop New Indexes in Crohn’s disease group has proposed the concept of “cumulative bowel damage” as an underlying mechanism for this phenomenon [36]. They postulate that ongoing inflammation results in irreversible damage that is cumulative over time, resulting in quicker progression and earlier manifestations of complications.

The results of the CALM trial support this hypothesis by comparing two medical treatment strategies in patients with active CD that were dependent on steroids but have not yet used any biologics [6]. A total of 244 patients were randomized between tight control of disease activity vs regular clinical management. They achieved this by using a similar step-up treatment strategy, but with different criteria for step-up. Patients in the tight control group were escalated more readily based on a stricter mix of clinical and laboratory criteria. A significantly higher proportion of patients in the tight control group achieved mucosal healing (46% vs 30%), which was the primary outcome of the study. Adverse events were equal between groups. Several other trials also showed results suggesting that earlier treatment with biologicals, such as certolizumab or adalimumab, may achieve better treatment outcomes in terms of remission rates [37, 38]. Similarly, two independent Canadian cohort studies showed that treatment with anti-TNF biologics within 2 years of diagnosis achieved better disease outcomes than delaying treatment for >2 years [39, 40]. One study showed fewer disease-specific and overall hospitalizations in the 5 years following the start of therapy [40], while both studies showed lower rates of surgery in the earlier anti-TNF group.

On the other hand, one should also be aware of the limitation of biologics. A recent post hoc long-term follow-up study of the TAILORIX trial showed that achieving long-term remission off-steroid does not ensure longer disease-free survival [41]. In this study, 95 patients were followed up for a median duration of 5 years. They compared 45 patients who had achieved sustained remission for >30 weeks to 50 patients who did not. Comparative analysis showed no difference between both groups in terms of frequency of major abdominal surgery, anal surgery, CD-related hospitalization, or the need for a new systemic treatment. Also, it is uncertain whether biologics do result in a decrease in the length of surgical resection once the surgery is indicated. De Groof *et al.* [42] showed in a retrospective cohort that, between 1999 and 2014, more biologics were prescribed to patients and that this was associated with an increase in time from diagnosis to operation. However, the length of the resected ileum did not change significantly over time with a median of 20.0 cm. Thus it seems that while early and tight medical treatment might achieve better disease control and relieve symptoms, prolonged medical management does not necessarily show benefit in the long term for disease progression or extent of surgery.

Another important factor to consider is that undue delay of surgery has been shown to increase post-operative risk. Iesalnieks *et al*. [23] examined the effect of a prolonged period between onset of clinical exacerbation unresponsive to medical treatment on the outcome of surgical treatment. They showed that a time between the onset of exacerbation and surgery of >5 months was associated with a higher number of organs involved in the inflammatory mass, more profound preoperative weight loss, and a higher rate of use of steroids or other immunosuppressive drugs. Patients in this group also had a higher post-operative risk of complications as indicated by a higher rate of post-operative septic complications (31% vs 13%, *P *=* *0.002).

### Combining medical and surgical treatment: biologics after surgery

There is a growing body of evidence that early initiation of biologicals after surgery might be beneficial for the disease course in CD. In a 2012 RCT, 31 patients after ileocolic resection were randomized to receive scheduled infliximab treatment (*n *=* *15) vs no infliximab (*n *=* *16) for 3 years [43]. At 1 and 3 years, 100% and 93% of patients in the infliximab group were in remission vs 69% and 56% in the control group (*P *<* *0.03), respectively. Additionally, the infliximab group achieved higher endoscopic remission at 1 year with 79% vs 19% (*P *=* *0.004).

A different multicenter international RCT published in 2016 randomized 297 patients after ileocolic resection to either infliximab or placebo for 200 weeks [44]. Use of infliximab showed a reduction in endoscopic recurrence (31% vs 60% at week 76, *P *<* *0.001) and a trend for less clinical recurrence (25% vs 15% at 2 years, *P *=* *0.1). A subsequent trial examined whether routine colonoscopy at 6 months after intestinal resection to guide treatment was of added value compared with clinical assessment alone [45]. The authors concluded that step-up treatment according to clinical risk with early colonoscopy is better than conventional therapy alone for the prevention of post-operative recurrent CD. Finally, a recent large retrospective cohort showed similar results [46]. In this study, 1,037 patients who underwent ileocolic resection for CD were included. A total of 278 patients received post-operative prophylactic biologics. Prophylaxis was initiated within 4 weeks in 35% of patients and between 4 and 12 weeks in the remainder of patients. The study found that early initiation of an anti-TNF agent within 4 weeks following surgery was associated with a reduction in the post-operative recurrence rate (HR = 0.61; 95% CI 0.40–0.93).

## Patients’ perspective

In CD, where the impact on patients’ lives and QOL can be paramount, it is important to also examine the opinion and experiences of patients regarding surgery. In 1994, 80 British patients were surveyed regarding the timing of their ileocolic resection for CD [47]. None of the 70 (88%) patients who replied would have preferred their surgery to have been later, while 74% would have preferred it to have been earlier. The median preferred time was 12 months earlier, with 95% from 7 to 18 months. Reasons given for earlier surgery were mainly the severity of symptoms prior to surgery (97%), the ability to eat normally (86%), and the feeling of well-being after surgery (62%). These results are confirmed by a recent systematic review examining the QOL after intestinal resections in patients with CD [19]. The reviews showed that surgery consistently increased health-related QOL and that patients described high satisfaction with their procedures. One of the included studies showed that post-operative scores of QOL improved to levels comparable to those of the general population and concluded that they believed this to justify early surgical intervention in many patients with symptomatic CD [48].

## Summary

CD can involve the entire gastrointestinal tract from the mouth to the anus and can lead to a constellation of symptoms. Disease location, severity, associated complications, and their impact on individual patients can vary significantly. As with most non-malignant diseases, the choice of surgery vs medical treatment is very much a patient’s personal preference under the guidance of the treating physician, except in extreme situations where surgery might be the only option.

Table 1 discusses the advantages and disadvantages of surgery, which need to be assessed in any determination of the relative role of surgical intervention over medical treatment. Different phenotypes might also well determine the medical vs surgical candidates in a particular situation. Given this context, comparisons of upfront medical vs surgical treatment are difficult. Table 2 lists the important findings about the timing of surgery for CD. The review of the literature clarifies some points. When surgery is chosen as the first-line treatment, a proportion of patients continue to do well on follow-up without the need for medical treatment. Upfront surgery can be performed safely even for acute disease when this is localized and can be expected to be associated with at least comparable and likely favorable intermediate and long-term outcomes compared with medical treatment. Patients who have undergone surgery and hence have the opportunity to assess their situation when treated medically vs surgically express a preference for an earlier decision for surgery. After surgical resection, the use of biologics as the immunosuppressive treatment reduces endoscopic and clinical recurrence (Table 3).

**Table 1.** Advantages and disadvantages of surgery in Crohn’s disease

Advantage	Disadvantage
Quicker relief of symptoms	Risk of post-operative complications, including stoma
Acquire certainty about diagnosis and presence of cancer	Not easily repeatable (adhesions, short-gut syndrome)
Reduction of the inflammatory burden to aid other modalities	Recurrence (especially if margins are positive for inflammation)
Improved quality of life	Needs skilled surgeon

**Table 2.** Most important findings regarding timing of surgery in Crohn’s disease

Event	Conclusion	Study type
Early surgery (vs initial medical management)	Early surgery is feasible and safe	RCT [27, 29]
	Provides at least similar quality of life	RCT [27, 29]
	Similar adverse events/complication rates to medical management	RCT [27, 29], retrospective study [32]
	Reduced risk of clinical recurrence	Retrospective study [24]
	Reduced risk of subsequent surgery	Retrospective study [25]
	Fewer immunosuppression requirements	Retrospective study [25, 26, 32, 34]
Surgery at a later disease stage (vs early surgery)	Higher risk of surgical complications	Retrospective study [23, 30, 31, 33]
	Higher rate of reoperations	Retrospective study [31]
	Higher risk of medical complications	Retrospective study [23, 31]
	More extensive surgery required	Retrospective study [23]

RCT, randomized–controlled trial.

**Table 3.** Most important findings about combining biologics with surgery

Event	Conclusion	Study type
Biologics prior to surgery	Quicker escalation using a mix of clinical and endoscopic criteria achieves better disease control	RCT [6, 37, 38]
	Long-term remission off-steroid does not ensure longer disease-free survival	RCT [41]
	Biologics delayed surgery, but does not reduce the extent of surgical resection needed	Retrospective study [42]
	In case of unresponsiveness, delaying surgery increases the difficulty and risk of surgery	Retrospective study [23]
Biologics after surgery	Early initiation of biologics after surgery can be beneficial for the disease course	RCT [43, 44], retrospective study [46]
	Routine endoscopic control and treatment accordingly can delay/prevent disease recurrence	RCT [45]

RCT, randomized–controlled trial.

## Recommendations

With the advancement of effective medical treatments for CD, a tendency has emerged to consider surgical treatment as the last resort. In some cases, this has resulted in long-drawn-out treatment schedules in which therapies are escalated and switched for long periods. When such schemes fail, patients are left sicker, less well nourished, and with more severe complications. A German prospective survey examined changes in surgical therapy of CD over 33 years [49]. It showed that with improved medical treatment, an increase in elective surgery compared with urgent surgery was seen. This was, however, associated with a significant increase in stenosis and ileus as indications for surgery, and more importantly also a significant increase in serious acute complications like free bowel perforations and peritonitis necessitating surgery. It is not surprising, therefore, that several clinicians and researchers have challenged this notion prompted by the emerging body of evidence discussed above [50–52].

Based on this, we have attempted to formulate recommendations that can help in guiding clinical management based on the evidence available. First, it is currently well established that early surgical resection in patients with limited disease that do not respond promptly to medical treatment is a feasible and safe option. Several guidelines are now recommending it as an equal therapeutic alternative to medical therapy [50, 53–55]. We feel that this recommendation can be extended to all patients who are not high-risk surgical patients (i.e. not the American Society of Anesthesiologist class IV or have extensive prior abdominal surgery) and who might have disease complications that are amenable to resection, such as strictures or limited penetrating disease. Care must be taken to get patients into a well-nourished state and preferably off steroids before surgery.

Patients with more complex penetrating diseases (fistula or abscesses) present a bigger therapeutic challenge. The presence of such complications is associated with a 3-fold increase in the risk of failure of medical treatment [56]. Similarly, surgery in such patients might require extensive resections increasing the post-operative risk of complications, diversion, and short-gut syndrome. For these patients, a multidisciplinary approach is necessary. Initial treatment with combined immunosuppressive regiments and frequent assessment of its effect is recommended [57]. If the disease becomes more limited due to medical treatment, surgery should be performed since total resolution with medical management alone is unlikely. If medical treatment does not yield the expected results, undue delays in surgery should be avoided and patients should be referred to experienced surgeons for treatment. After surgery, early medical prophylaxis using biologicals, especially in patients with risk factors for progressive disease, is highly recommended based on current evidence.

Patients with predominantly fibrotic or stenotic presentations should be referred to surgery or endoscopy as the preferred treatment since no effective medical treatment modality exists [58]. In these cases, the use of biologics might delay inevitable surgery, which can be detrimental to patients’ outcomes. Finally, in patients for whom surgery is not feasible, such as high-risk patients or those with risk for short-gut syndrome, a strategy of maximal medical management can be the only feasible approach.
